# Perceptual Validation of Nonlinear Postural Predictors of Visually Induced Motion Sickness

**DOI:** 10.3389/fpsyg.2020.01533

**Published:** 2020-07-15

**Authors:** Max A. Teaford, Henry E. Cook, Justin A. Hassebrock, Robin D. Thomas, L. James Smart

**Affiliations:** Department of Psychology, Miami University, Oxford, OH, United States

**Keywords:** perception, categorization, posture, motion sickness, nonlinear measures, sort task

## Abstract

Virtual reality (VR) technology has become increasingly prevalent in our society and has been used for a myriad of applications ranging from psychotherapy to training members of the military. However, one issue that arises from the use of VR is motion sickness, thus making predictors and indicators of motion sickness desirable. To date, a number of indicators of motion sickness have been derived based on nonlinear characteristics of human motion recorded using motion capture systems. While it is known that nonlinear measures can be used to predict motion sickness, it is not known whether people are perceptually sensitive to these particular nonlinear parameters. The aims of this study included establishing whether individuals consistently sort phase plots of sick and well individuals’ postural motion without being explicitly told to do so; determining what nonlinear movement parameters could be used to represent these judgments; and assessing the stability of nonlinear measures found to be successful at predicting motion sickness by Smart et al. (2014). Through two methods of analysis (perceptual and quantitative), this research demonstrated that participants can indeed sort the graphic depictions of sick and well participants’ postural motion and seem to be perceptually sensitive to nonlinear parameters (normalized path length, path length, elliptical area) that are known to be predictive of motion sickness.

## Introduction

Virtual reality (VR) has become an increasingly commonplace technology largely due to declining costs to purchase commercial systems and the wide array of applications for this technology. Some applications for this technology include exposure therapy (Carl et al., 2019), training for the military (Bowman and McMahan, 2007), and entertainment (e.g., video games; Zyda, 2005). However, motion sickness stemming from virtual environment use is an ongoing issue across these settings. The incidence of motion sickness has significant implications for the continued viability of this technology. Given this, developing the ability to predict motion sickness in order to mitigate its prevalence through intervention and training is important.

One potential indicator of motion sickness that has emerged in recent years is postural motion. It has been found that changes in postural motion precede motion sickness and can also be used as a predictive tool (Stoffregen and Smart, 1998; Yokota et al., 2005; Smart et al., 2007; Otten and Smart, 2009; Chang et al., 2012; Palmisano et al., 2018). Evidence for this claim has been found with both physical situations (moving room: Smart et al., 2002; naval craft: Stoffregen et al., 2013) and virtual stimuli (such as a military simulator, Stoffregen et al., 2000; as well as a virtual moving room: Villard et al., 2008). However, basic descriptive means of quantifying posture (e.g., variability, velocity, or range) have yielded inconsistent results, suggesting that these measures may not be capturing the key aspects of postural motion that would allow for consistent prediction. Recently, it was discovered that nonlinear measures of kinematic data, indicative of movement strategies, not only precede motion sickness (Villard et al., 2008; Stoffregen et al., 2010), but can be used to predict whether a person will remain well or become motion sick (Smart et al., 2014; Hadidon, 2016). In particular, it was found that participants who remain well tend to exhibit less complex but more temporally flexible movement strategies than those who become motion sick (Smart et al., 2014; Cook et al., 2018).

**FIGURE 1:**
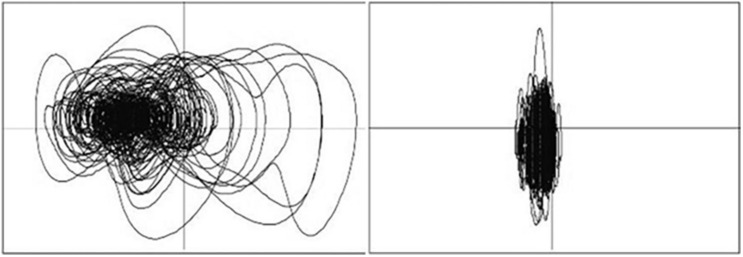
Examples posture phase plots (anterior–posterior position × anterior–posterior velocity). When presenting these types of figures, people can readily distinguish between “sick” **(left)** and “well” **(right)** plots when prompted.

While it is known that quantitative measures of posture can indeed predict motion sickness, it has yet to be seen if people can detect differences in postural strategies (which in turn are indicative of motion sickness) using the same changes that quantitative measures known to be sensitive to postural instability and subsequent motion sickness appear to exploit. Previous research has demonstrated that individuals can judge relative phase from visual depictions of kinematic data (Bingham et al., 1999; Zaal et al., 2000). These judgments seem to be a function of quantitative movement parameters (i.e., mean relative phase; Zaal et al., 2000). There are also anecdotal reports of people having the ability to accurately classify motion sickness (Smart, 2010). For example, when individuals are shown phase plots such as those depicted in Figure 1, they can consistently and accurately select the plot from the sick individuals (which in this case is the phase plot on the left). In addition, it has been casually observed that when conducting motion sickness studies in our laboratory, the research assistants began to recognize potentially motion-sick participants by changes in their observable movements. In conjunction, these two lines of evidence suggest that participants should be able to differentiate between visual depictions of sick and well participants’ movement data and may be able to detect characteristics of it that are exploited by quantitative measures.

The anecdotal observations regarding people’s ability to discriminate healthy and problematic postural motions also suggest that movement could be a “trainable” source of information; the current study is the first formal empirical study designed to examine what underlies this ability. Cook et al. (2018) were able to show that participants physically respond differently to motion of healthy and motion-sick participants, supporting the informal observations of our research assistants’ reactions to potentially motion-sick participants. However, this study did not assess perceptual phenomena, focusing instead on behavioral reactions to the motion stimuli.

Other research studies have been able to demonstrate that participants are able to discriminate complex categories of various types, including nonlinear relations, despite not being able to state explicitly what the underlying membership or category rule was (e.g., Gibson and Gibson, 1955; Thomas, 1998; Ashby et al., 2003; Guest and Lamberts, 2010). This suggests that participants can perceive and act on complex relations even if they cannot explicitly verbalize what they are acting on. One particularly relevant example that supports this possibility comes from a classic study by Gibson and Gibson (1955), who used stimuli that looked like scribbles (while still having structure) and found that participants could sort them accurately despite not knowing what the scribbles were or what they potentially represented. Gibson and Gibson (1955) asserted that the ability of participants to successfully discriminate among seemingly random patterns reveals that the role of experience (perceptual learning) is to aid in fine tuning the perceptual systems to take advantage of information present in the environment, a process they termed *differentiation*. In the categorization literature, there are many examples of participant’s ability to create and sort items into aggregate groups (e.g., food, colors, personalities, geometric forms; Purcell and Thomas, 2007; Bimler, 2013; Ritter and Preston, 2013). Sorting methodologies are employed often in marketing and business to help discover how people understand and relate products (Varela and Ares, 2012). This study will be the first to use the sort-and-merge methodology to validate perceptually the efficacy of nonlinear movement parameters shown to predict motion sickness.

## The Current Study

In the present study, we employed a sorting task in which participants were asked to sort phase plots of sick and well participants’ postural sway data while remaining naive to the origin of the phase plots. Participants were assigned to one of three conditions that varied in sorting constraints. The aims of the present study were the following; (1) establish whether or not individuals can indeed consistently sort phase plots of sick and well individuals without being explicitly being told to do so, (2) determine if more constrained task sorting instructions change sorting judgments, (3) determine what nonlinear movement parameters could be used to represent these judgments, and (4) assess the stability of nonlinear measures found to be successful at predicting motion sickness by Smart et al. (2014). This was achieved by performing a secondary analysis of the data Stoffregen and Smart (1998) and Smart et al. (2002), which were used to create the phase plots used in the present study.

## Materials and Methods

### Participants

Ninety-three undergraduate students from a large Midwestern state university participated in the present study for course credit. Demographic information was not collected, as those parameters should not have influenced the task. However, the samples did reflect the makeup of the general psychology undergraduate population: 60% female, 80% Caucasian, and age range of 18 to 22 years. Thirty-six participants were in the free-choice condition, 30 in the forced-scale condition, and 27 in the binary-choice condition. The data for each condition were collected during separate semesters to ensure that independent samples were obtained. Participants gave informed consent in accordance with the Declaration of Helsinki but were not told the purpose of the study or what the stimuli represented until they completed the task. The study protocols were approved by the Miami University Research Ethics and Integrity Office’s review board (E00432).

### Stimuli

**FIGURE 2:**
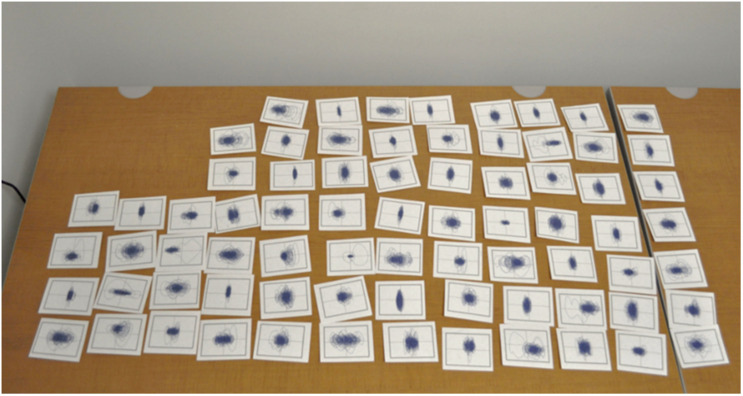
Phase plot (all plots drawn to same scale) setup, cards were laid out prior to participants’ arrival.

Seventy-four postural motion phase plots composed of anterior–posterior (AP; horizontal axis) position versus AP velocity (vertical axis) were created using postural data from Stoffregen and Smart (1998) and Smart et al. (2002) and were printed on 3 × 4-inch cards. Twenty-eight of the cards represented motion from participants who became sick. Each plot represented 10 min of motion data while being exposed to complex optic flow with amplitude and frequency characteristics that approximate that generated by human postural motion (see Stoffregen and Smart, 1998 and Smart et al., 2002). Importantly, all of the phase plots represent data collected before explicit reports of motion sickness by participants in the original studies. All phase plots were drawn to the same scale. There were no indications of what the cards represented (cards were labeled on the back for the experimenter; the labeling conventions were not meaningful for the participants). The cards were shuffled and then spread out, in a pseudorandom order on a table prior to the participants’ entrance (Figure 2).

### General Procedure

Upon providing informed consent, participants were then taken to a separate room where they were presented with seventy-four 3 × 4-inch cards with pictures of phase plots on them. No participants (in any condition) were told what the plots represented, and the axes in the plots were not labeled. Participants were asked to divide the cards into categories using procedures dictated by the condition the participant was in as described below. These participant-created categories were attached to index cards using a paper clip to keep the member plots together. Participants were either told to sort cards into as many groups (≥2) as they wanted (free-choice condition) based on perceived similarity; into 10 groups (forced-scale condition) based on perceived “healthiness” (with 1 being least healthy, 10 being most healthy), with at least one card being placed in each category; or into two groups (binary-choice condition–healthy, unhealthy). Participants were never asked to make judgments or sort cards based on whether the cards indicated motion sickness. Once the cards were sorted, participants were then asked to merge the categories based on perceived similarity; thus, if a participant’s first and third categories were perceived as being similar, these categories were combined. This process continued until there was only a single category left. Both the initial card memberships in each category and the order in which participants combined categories were recorded, and these data were analyzed using a multidimensional scaling procedure (described in the following section). Once the sort-and-merge process was completed, participants were debriefed and allowed to ask questions about the study, after which they were given course credit and could leave.

### Data Analysis

The motion capture data from Smart et al. (2002) and Stoffregen and Smart (1998; used to create all of the phase plots) were reanalyzed using a custom MATLAB script, which computed path length, normalized path length, elliptical area, and normalized sample entropy (described in Smart et al., 2014), as well as computing Hurst exponents, which are a measure of self-similarity across time scales (Littman, 2011). In principle, Hurst exponents and sample entropy are comparable measures. For the purposes of statistical analysis, the Hurst exponents (range, 0–1, with 0.5 indicating random noise) were normalized (using the inverse normal function in Microsoft Excel; new range, −1 to 1, with 0 indicating random noise). Previous research by Smart et al. (2014) found the aforementioned measures to be sensitive to motion sickness. These measures were analyzed using stepwise discriminant analysis (SDA) as a secondary analysis of the original data. This was done to assess whether these nonlinear measures were able to predict motion sickness, as well as (or better than) the measures employed by Smart et al. (2002).

**FIGURE 3:**
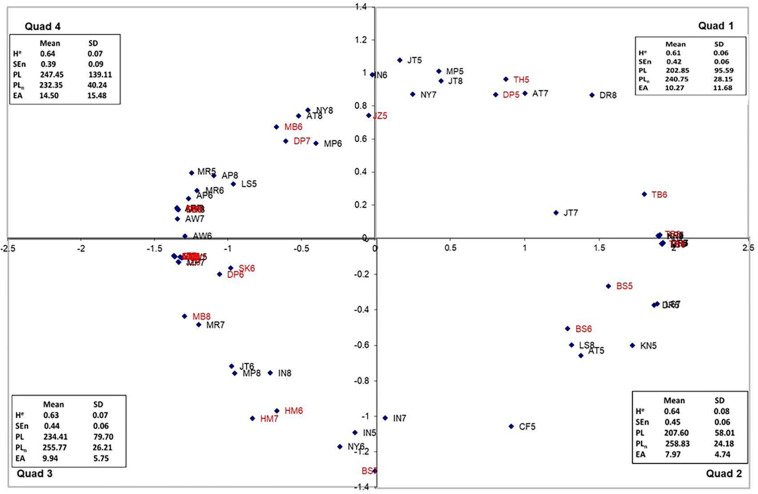
The dimensional map for the free-choice condition, Quadrants 2 and 3 represent the “tails” of the map and are psychologically (perceptually) most distant. Trials from motion-sick participants are in red.

The similarity and patterns formed by the data were examined using a few steps. For the first step, a metric of similarity among the postural data figures was constructed based on the participants’ category groups and merges. Custom software was used to create the distances (see the Supplementary Material Section 1 for details about the algorithm used). This software read the data from an Excel file with the participants’ initial sort data and an Excel file with the participants’ merge data. It then created two matrices, a similarity matrix for every participant, and a matrix containing the summed data across participants. Multidimensional scaling analyses (MDS; Alscal; IBM Corp., Released 2011, IBM SPSS Statistics for Windows, version 20.0, IBM Corp., Armonk, NY, United States) were used to convert these similarities into a spatial map featuring the objects. The SPSS analysis assumed geometric distances between points and was set to generate a two-dimensional solution. Using this coordinate map, a picture of the object’s important attributes and relationships to one another may be revealed. The distance between points indicates how similar the stimuli were perceived to be by the participants (Smith, 1995; Braun, 2012). Spatial maps of all three experimental conditions were made (Figures 3, 5, 7). The author chose to label the quadrants in a clockwise fashion for data management purposes because directionality does not matter.

In order to examine the relationship between the nonlinear motion parameters and participants’ sorting dimensions, a path analysis was used. The path analysis was conducted using the seventh edition of Mplus (Muthén and Muthén, 2017). Prior to analyzing the data, the data were screened for outliers, missing variables, and multivariate normality. Mahalanobis *D*^2^ was used to screen the data for multivariate outliers, but no multivariate outliers were found. The percentage of data missing was 0%. Each variable was checked for normality using histograms–all variables were found to exhibit departures from normality. Therefore, Mplus’s MLM estimator was used because it is robust to non-normality.

The criteria for acceptable model fit were determined *a priori*. In order for the model to be acceptable, it must have a χ^2^ test that was non-significant, a CFI and TLI value of 0.95 or greater, an RMSEA value of 0.08 or less, and an SRMR of 0.08 or less. Failing to meet any of these criteria is grounds for model respecification. For the exact values of the fit indices and path coefficients, see the Supplementary Material.

## Results

The patterns formed by the data from all three experiments appear remarkably similar; all form a “u” shape within the four quadrants spatial maps (Figures 3, 5, 7). This shape is an artifact of the analysis; items at each end of the “u” are actually not close to the items on the other end despite the fact the quadrants are next to each other (Figure 3). For example, in the binary-choice plot (Figure 7), bs7 (Q4) and cf5 (Q1) are on opposite ends of the spectrum, as far apart from each other as possible. Because direction is irrelevant in this type of analysis, the fact that the pattern points downward in the free-choice condition and upward in the binary and forced-scale conditions is not a meaningful difference.

### Stimuli Movement Parameters

An SDA was performed in order to determine if the nonlinear movement parameters utilized in Smart et al. (2014) could be used to accurately classify motion from sick and well participants in this dataset (from Stoffregen and Smart, 1998 and Smart et al., 2002). In addition, we included the stimuli coordinates derived from the sort/merge task in the current study. The values included in the analysis were sorting Dimension 1, sorting Dimension 2 (from the current study), sample entropy, elliptical area, path length, normalized path length (used in Smart et al., 2014), and the Hurst exponent. One function was found that significantly differentiated the groups and was consistent across all of the conditions, Wilks λ = 0.56, χ^2^(3) = 40.82, *p* < 0.01, canonical correlation (*R*_c_) = 0.66, and accounted for 44% of the variance in group membership. This function was found to correctly classify 79.7% of the cases. Using the cross-validation method described in Smart et al. (2002), it was found that 78.4% of the participants were still classified correctly. This level of accuracy is comparable with that found by Smart et al. (2002).

The structure matrix of the variables included in the function and group centroids for the function can be seen in Tables 1, 2. According to the structure matrix, the first and only function included normalized path length, path length, and sample entropy, suggesting the function encompasses the complexity of movement. The group centroids suggest that the function that was found is elevated in sick individuals and lower in healthy individuals. Interestingly, the function found by the discriminant analysis was very similar to the results of the path analysis conducted on the sorting data dimensions. The implications of this finding will be elaborated upon in the *Discussion*. The sorting dimensions did not contribute to this analysis; we believe this to be because, treated independently, which SDA does, the dimensions do not uniquely specify a given stimuli (i.e., several stimuli can have the same *x* coordinate but different *y* coordinates).

**TABLE 1:** The structure matrix resulting from the discriminant analysis performed on the motion data used as stimuli.

Measure name	Structure matrix coefficient
Normalized path length	–0.466
Path length	0.430
Sample entropy	–0.200

**TABLE 2:** Centroids for the sick and well groups.

Group	Centroid
Well	–0.682
Sick	1.120

### Free-Choice Condition

#### Multidimensional Scaling

As stated above, multidimensional scaling (Alscal) was performed using the sorting data from the participants in the free groups condition. The perceptual map produced from the analysis of the participants’ judgments can be seen in Figure 3, along with the mean and standard deviation of each nonlinear measure of the plots in that quadrant. The stress index for this model was 0.07012, and the R-squared (variance accounted for by model) (RSQ) value was 0.98368, both of which suggest that the perceptual map matches the observed data well.

#### Path Analysis

**FIGURE 4:**
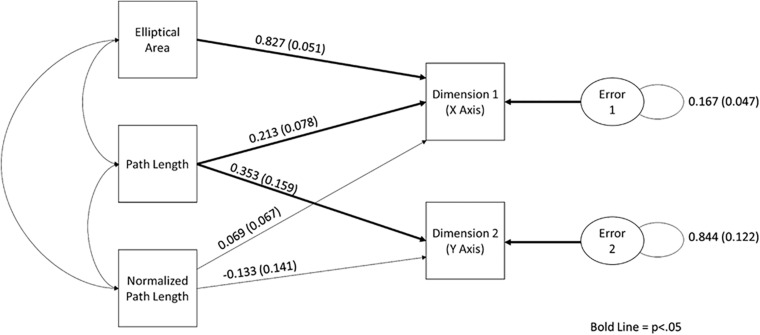
The path diagram demonstrating the relationship between elliptical area, path length, normalized path length, and sorting dimensions. Standardized path coefficients and standard error are reported on this figure.

In order to establish the relationship between the nonlinear postural characteristics and sorting dimensions, a path analysis was conducted. As stated above, a path analysis was conducted in Mplus (version 7; Muthén and Muthén, 2017) using the MLM estimator (see Supplementary Table S1 for the covariance matrix). The expected relationships between manifest variables can be seen in Figure 4. It was found that the model was overidentified, and the global fit of the model was acceptable, χ^2^(2) = 0.80, *p* > 0.05. Furthermore, all of the local fit indices suggest that the model fits well (see Supplementary Table S2 for the fit index values).

Upon examination of the path coefficients, it was apparent that path length (*p* < 0.01) and elliptical area (*p* < 0.01) were a significant predictor of Dimension 1, whereas only path length was a significant predictor of Dimension 2 (*p* < 0.01). Normalized path length was not found to be predictive of either dimension in this condition. It should be noted that all three measures were needed to produce a stable model. See Figure 4, for the standardized path coefficient estimates and their standard error (see Supplementary Table S3 for all of the path coefficients and their exact *p* values).

### Forced-Scale Condition

#### Multidimensional Scaling

**FIGURE 5:**
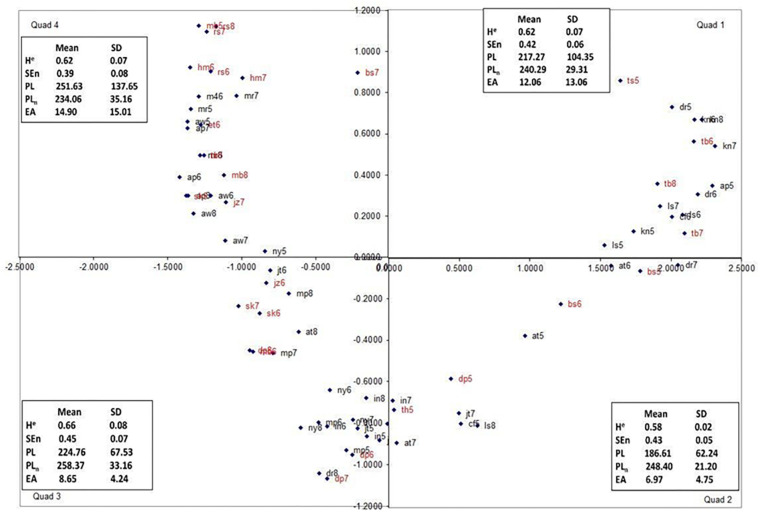
The dimensional map for the forced scale sorting condition, Quadrants 1 and 4 represent the “tails” of the map and are psychologically (perceptually) most distant. Trials from motion-sick participants are in red.

As stated above, multidimensional scaling (Alscal) was performed using the sorting data from the forced-scale condition. The perceptual map produced from the analysis of the participants’ judgments can be seen in Figure 5, along with the mean and standard deviation of each nonlinear measure of the plots in that quadrant. The stress index for this model was 0.06664, and the RSQ value was 0.98402, both of which suggest that the perceptual map matches the observed data well.

#### Path Analysis

**FIGURE 6:**
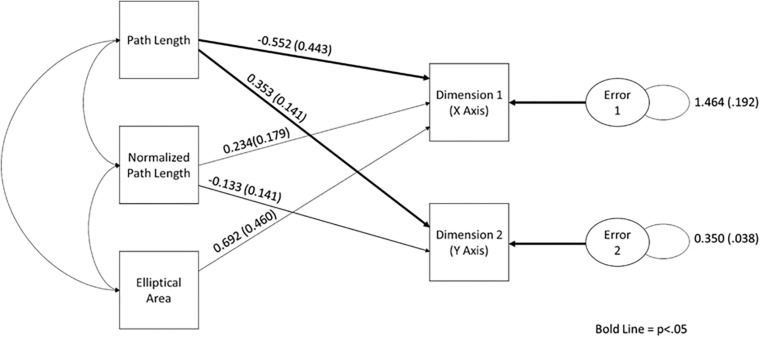
The path diagram demonstrating the relationship between elliptical area, path length, normalized path length, and sorting dimensions. Standardized path coefficients and standard error are reported on this figure.

As stated above, a path analysis was conducted in Mplus (version 7; Muthén and Muthén, 2017) using the MLM estimator (see Supplementary Table S4 for the covariance matrix). The expected relationships between manifest variables can be seen in Figure 6. It was found that the model was overidentified, and the global fit of the model was acceptable, χ^2^(2) = 1.9, *p* > 0.05. Furthermore, all of the local fit indices suggest that the model fits well (see Supplementary Table S5 for the exact fit index values).

Upon examination of the path coefficients, it was apparent that only path length was predictive of Dimension 1 (*p* < 0.05). Path length was also the only variable that was a significant predictor of Dimension 2 (*p* < 0.05). Neither elliptical area nor normalized path length was found to be a statistically significant predictor of either dimension in this condition. It should be noted that all three measures were needed to produce a stable model (see Supplementary Table S6 for all of the path coefficient values and exact *p* values). See Figure 5, for the standardized path coefficient estimates and their standard error.

### Binary-Choice Condition

**FIGURE 7:**
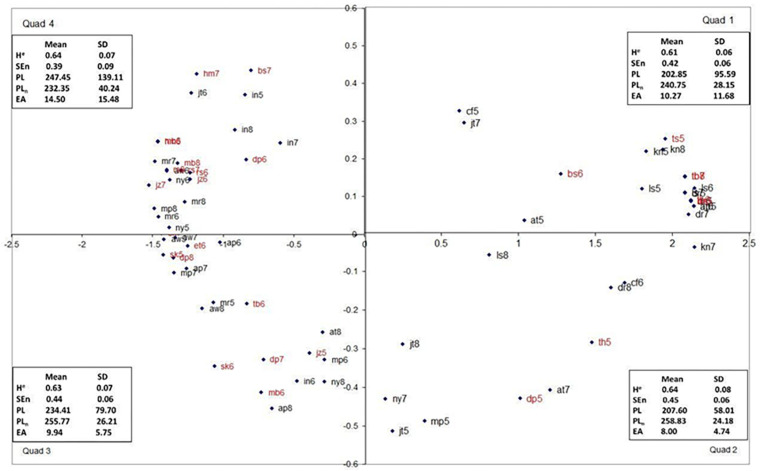
The dimensional map for the binary-choice sorting condition, Quadrants 1 and 4 represent the “tails” of the map and are psychologically (perceptually) most distant. Trials from motion-sick participants are in red.

As stated above, multidimensional scaling (Alscal) was performed using the sorting data from the binary-choice condition. The perceptual map produced from the analysis of the participants’ judgments can be seen in Figure 7, along with the mean and standard deviation of each nonlinear measure of the plots in that quadrant. The stress index for this model was 0.01863, and the RSQ value was 0.99901, both of which suggest that the perceptual map matches the observed data well.

**FIGURE 8:**
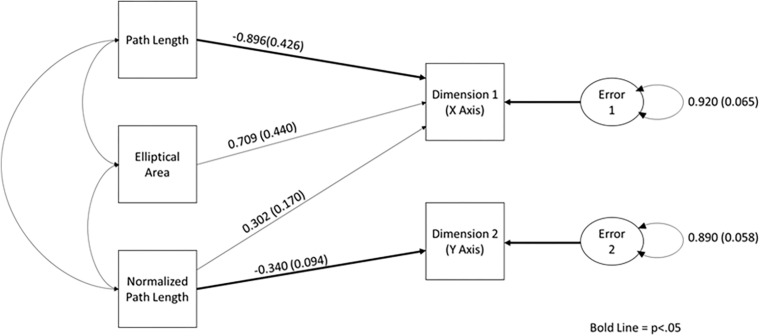
The path diagram demonstrating the relationship between elliptical area, path length, normalized path length, and sorting dimensions. Standardized path coefficients and standard error are reported on this figure.

As stated above, a path analysis was conducted in Mplus (Muthén and Muthén, 2017) using the MLM estimator (see Supplementary Table S7 for the covariance matrix). The expected relationships between manifest variables can be seen in Figure 8. It was found that the model was overidentified, and the global fit of the model was acceptable, χ^2^(3) = 2.30, *p* > 0.05. Furthermore, all of the local fit indices suggest that the model fit well (see Supplementary Table S8 for the fit index values).

Upon examination of the path coefficients, it was apparent that path length was a significant predictor of Dimension 1 (*p* < 0.05), and normalized path length was predictive of Dimension 2 (*p* < 0.01). However, neither elliptical area nor normalized path length individually predicted Dimension 1. It should be noted that all three measures were needed to produce a stable model. See Figure 8, for the standardized path coefficient estimates and their standard error (see Supplementary Table S9 for all of the path coefficients and their exact *p* values).

## Discussion

The current study sought to employ a common category formation paradigm (sort/merge task) to examine whether there are perception-based indices of postural motion that can be used to validate/support quantitative indices that have been used in the literature (Smart et al., 2014; Hadidon, 2016; Cook et al., 2018). Through two methods of analysis (perceptual and quantitative), this research demonstrates that there is a viable set of (nonlinear) measures that can be used to identify and predict motion sickness. In addition, we sought to determine if more constrained task sorting instructions would change sorting judgments. The results of the present study confirm that individuals can consistently sort phase plots without explicitly having knowledge of what those plots represent. What is important about this outcome are the parameters that the participants seemed to exploit when completing the task map almost directly onto the same nonlinear measures shown to be effective in other studies (and in the current study). Furthermore, task constraints did indeed change the participants’ sorting task performance, although overall distributions of the plots were very similar across conditions. The change in instructional constraints also produced increasingly constrained use of movement parameters (i.e., path length, normalized path length, and elliptical area) for sorting judgments. This finding is made even more interesting by its similarity to the function derived using discriminant analysis to sort data into sick and well groups using movement parameters.

In order to understand better participants’ sorting judgments, multidimensional scaling (Ascal) was used on the data from each sorting condition. As can be seen in Figures 3, 5, 7, the multidimensional scaling maps are remarkably similar across the three conditions. Based on examination of the stimuli from each quadrant, Dimension 1 (the *x* axis) appears to be spread of the phase plot, with higher positive numbers indicating more spread phase plots. Dimension 2 (the *y* axis) appears to reflect complexity of the phase plots, with higher positive numbers indicating more complexity. Based on the model statistics (low stress and high RSQ), in all of the models derived using multidimensional scaling, these indices seem to be the best descriptors of the changes that we observed across the maps. Interestingly, the sick individuals and well individuals during later trials (who often resemble participants who are sick) were more prevalent in Quadrants 3 and 4 of the multidimensional scaling perceptual map, indicating that they had high levels of phase plot complexity and low (Quadrant 4) or high (Quadrant 3) levels of phase plot spread. This reflects trends that were observed by Smart et al. (2002) as well as Hadidon (2016), suggesting that there are reliable characteristics that indicate non-optimal movement patterns.

**FIGURE 9:**
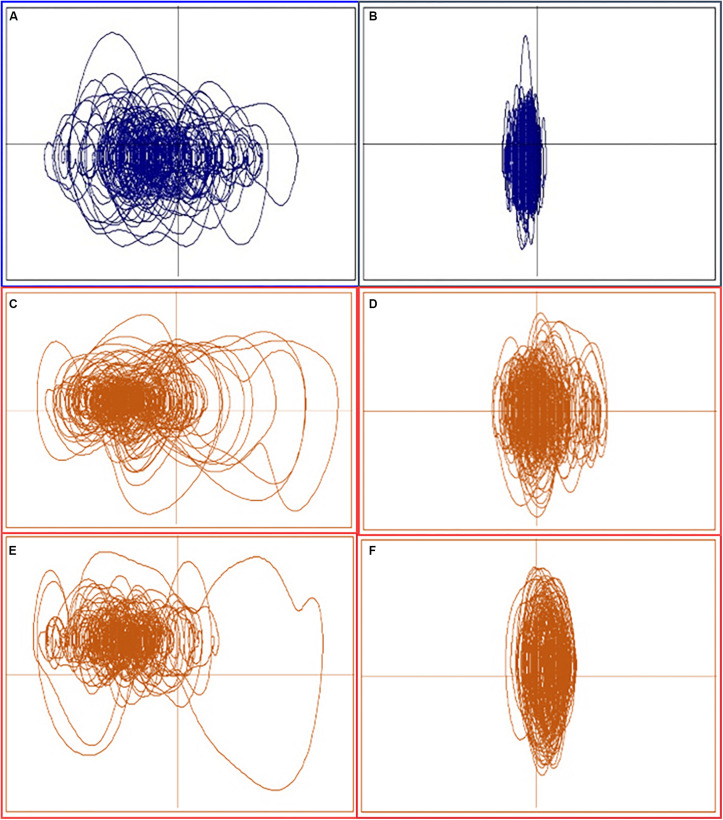
Representative phase plots at the extremes of each dimensional map. Left panels **(A,C,E)** are from Quadrant 3 (free choice)/4 (forced scale, binary choice), and right panels **(B,D,F)** are from Quadrant 2 (free choice)/1 (forced scale, binary choice). Plots in blue **(A,B)** are from well participants.

It is intriguing that the pattern generated by the participants in this study reflects the general idea that there may be an optimal level of variability (cf., Stergiou et al., 2006) and that exceeding or falling short of this can lead to problems. This is also consistent with Riccio and Stoffregen (1991), who suggested that instability could be exhibited in multiple manners (not only more movement, but also qualitatively different movement). In this case, we saw that the majority of motion-sick plots were clustered at the two extremes of the perceptual maps in all three conditions (see Figure 9, for representative plots). In addition, these extremes seem to represent trials that exhibited either too much variability/complexity or too little and lend empirical support to the idea that too much (“hypercontrol”) or too little (“hypocontrol”) regulation can lead to suboptimal outcomes such as motion sickness (Smart and Smith, 2001) or simply failing to maintain the intended behavior (Stergiou et al., 2006). This type of dual unstable regions was also noted by Chagdes et al. (2016), who noted in their model that postural movement outside of the stable region exhibited either rigid oscillatory characteristics or noisy (chaotic) characteristics.

In order to determine if participants were using different aspects of the motion to make sorting judgments, path analysis was used on the data from each condition separately. Based on the movement parameters that could be seen in the phase plots, a model in which path length and normalized path length predicted Dimensions 1 and 2, and elliptical area was predictive of Dimension 1. All models were found to have acceptable fit. Furthermore, as task instructions became increasingly constrained, so did the movement parameters that were used to make sorting judgments. As can be seen in the path diagram for the free-choice condition, elliptical area and path length were predictive of Dimension 1, and only path length was predictive of Dimension 2. In the forced-scale condition, only path length was predictive of Dimensions 1 and 2. In the binary-choice condition, path length was found to be predictive of Condition 1, and normalized path length was predictive of Dimension 2. Furthermore, the function derived using discriminate analysis was very similar to the statistically significant paths in the models. In essence, not only were movement parameters predictive of sorting judgments, but also the parameters used became more constrained as the task instructions became more constrained.

The fact that participants could sort phase plots of sick and well individuals consistently is not a trivial finding, particularly because they were never explicitly asked to do so. In light of the anecdotal reports of individuals being able to detect if someone is becoming ill by how they are moving (in the absence of a phase plot), this ability to detect structural patterns (for good or ill) seems to extend beyond static patterns (see, for example, Cook et al., 2018). Several studies (in other contexts) lend support to this possibility (Bingham et al., 1999; Zaal et al., 2000).

The findings of this study have several important implications for the development of VR. Virtual reality has frequently been associated with motion sickness, thus making an algorithm that can detect motion sickness before an individual is aware of it is highly desirable. Previous work by Smart et al. (2002) revealed that linear measures of postural sway (variability, velocity) were predictive of motion sickness, although not consistently. The nonlinear measures employed in this study seem to allow for more consistent classification and prediction of sickness, as they have been able to classify differences in well and motion-sick participants (Smart et al., 2014; Cook et al., 2018), as well as make predictions both *post hoc* (current study—using data collected 20 years ago) and in real time (Hadidon, 2016). The nonlinear measures also accounted for more of the variability in the data (44%) than the linear measures used in Smart et al. (2002; 31% variance accounted for). Based on the results of the present study, people also seem to be using nonlinear characteristics to classify phase plot cards of sick and well participants into categories that suggest a progression from useful to suboptimal movement patterns (strategies). This latter point is intriguing because it suggests that these nonlinear differences are not only something that can be detected, but used to make judgments about behavioral states in general and progression toward instability in particular.

The present study has a couple potential limitations. One potential limitation was that stimuli used did not allow participants to see the time course of the movement. This is reflected by the fact that the measures that are time dependent (Sample entropy and Hurst) did not contribute to the path analysis, but did contribute to the discriminate analysis (sample entropy). However, the fact that participants seemed to be using the same structurally based, nonlinear parameters (path length, normalized path length, elliptical area) that can be used to predict motion sickness suggests comparable results may be obtained using dynamic stimuli. Another limitation was that participants were not told what the phase plots were from and were never explicitly told to make judgments about motion sickness (the closest was “healthy” vs. “unhealthy”). This was done purposely to elicit “unbiased” categories, but may have increased variability in judgments. Knowing what the phase plots represented may have an impact on judgments.

Based on the data in the present study, it would seem that it may be possible to train people recognize non-optimal postural movements and potentially intervene before someone begins experiencing motion sickness symptoms. This could be achieved by some form of exposure training–presenting people with exemplar plots and having them sort based on the exemplars and then repeating the process until the person learned to detect the relevant traits. Machine learning techniques could also be applied to this type of data. Fortunately, these are possibilities that can be addressed through further research; the key finding is that these perceivable movement changes precede motion sickness and can provide early opportunities for prevention.

In summary, the results of the present study suggest that people are capable of detecting nonlinear aspects of postural phase plots that allow them to discriminate between motion generated by motion-sick and well participants. This ability to sort postural data is on par with quantitative methods and seems to be exploiting the same properties of the sway data, suggesting that nonlinear changes are not only a perceivable but also reliable indicator of behavioral states.

The data and stimuli for this research are available through Miami University Scholarly Commons: (Teaford et al., 2019).

## Data Availability Statement

The datasets generated for this study are available on request to the corresponding author.

## Ethics Statement

The studies involving human participants were reviewed and approved by Miami University Institutional Review Board. The patients/participants provided their written informed consent to participate in this study.

## Author Contributions

LS and RT developed the study concept. All authors contributed to the study design, drafted the manuscript, and approved the final version of the manuscript for submission. RT created the code to quantify the similarity ratings from the sort/merge task. HC and LS performed testing and data collection. MT, HC, JH, and LS performed the data analysis and interpretation.

## Conflict of Interest

HC is currently employed by the Vanguard Group, Inc. At the time the research was performed, Dr. Cook was student at Miami University and had no conflicting commercial or financial relationships. The remaining authors declare that the research was conducted in the absence of any commercial or financial relationships that could be construed as a potential conflict of interest.
