# Supplementary material for: Perceptual Validation of Nonlinear Postural Predictors of Visually Induced Motion Sickness
Source: Front Psychol. 2020 Jul 15;11:1533. doi: 10.3389/fpsyg.2020.01533 (PMC7375200; doi:10.3389/fpsyg.2020.01533)
Supplement: Supplementary file 1 [file Data_Sheet_1.docx]

**Supplemental Content**

**Section 1: MDS Software Algorithm Explanation**

At each stage of the sort-and-merge process, the collection of clusters is called a partition step beginning with the initial sort as partition step 1. For each participant *k* and for each stimulus pair *i* and *j*, we compute a proximity value, *p_ij_^(k)^* which is defined as the total number of partition steps (= merge stages + initial sort) in which the *i*-th and *j*-th stimuli are grouped together divided by the total number of partition steps (merge stages + initial sort) generated by that participant. For example, suppose eight stimuli are numbered 1, 2, 3, 4, 5, 6, 7, 8 and participant *k* produces the following partitions:

*Initial sort 1, 3, 5 | 2, 4 | 7, 8 | 6* (That is, stimuli 1,3, and 5 are sorted into Category 1, stimuli 2 and 4 are in category 2, stimuli 7 and 8 are in Category 3 and stimulus 6 is in the singleton Category 4. )

*Merge 1* 1, 3, 5, 7, 8 | 2, 4 | 6 (That is, Category 1 and 3 were merged)

*Merge 2* 1, 3, 5, 7, 8, 6 | 2, 4 (That is, old Cat. 1 & 3 merged with Cat. 4)

*Merge 3* all stimuli put together

The total number of partition steps for this subject is 4 = 1 initial sort + 3 merge steps. For stimuli 3 and 5, the number of sorting partition steps for which these stimuli were grouped together is 4 since they were grouped in the initial sort. Hence, *p_35_^(k)^* = 4/4 = 1. For stimuli 3 and 8, the number of sorting steps for which they were together is 3 since they were combined at the first merge step. Hence, *p_38_^(k)^* = 3/4. For stimuli 1 and 2, the number of sorting partitions for which they were together is 1, the final merge. Hence, *p_12_^(k)^* = 1/4. For each participant, an object × object matrix of these proximity values can be constructed in this manner. To obtain the overall group dissimilarity matrix, we averaged the entries of all participant proximity matrices and subtracted these cell averages from the total number of participants. That is, each cell *i, j* in the final group dissimilarity matrix = $n-\frac{\sum_{k} p_{ij}^{k}}{n}$= where n = number of participants. Proximities were converted to dissimilarities because the MDS procedure in SPSS expects the data to be dissimilarities. Any reverse monotonic transformation to the proximities will work as the scaling procedure only assumes ordinal scale of measurement for the data.

**Section 2: Information About the Path Analyses**

Table S1. *Covariance matrix for the variables included in the Free Choice condition model.*

| DIM1 DIM2     PATHL ELIP   PATHN |
| --- |
| DIM1            0.006  DIM2           0.115 62.163  PATHL        0.002 0.228         0.006  ELIP             2.378 31.133      0.599 1186.660  PATHN        0.022 -1.914       -0.015 9.873    1.702 |

Note: DIM1 = Dimension 1, DIM2 = Dimension 2, ELIP = Elliptical Area, PATHL = Path Length and, PATHN = Normalized Path Length

Table S2. *The values of the fit indices for the Free Choice condition model.*

| Fit Index                                     Fit Index Value                  Fit Indicated |
| --- |
| RMSEA                                            0.000                  Excellent Fit  CFI                                                   1.000              Excellent Fit  TLI                                                   1.000              Excellent Fit  SRMR                                              0.023                   Close Fit |

Table S3. *Standardized and unstandardized path coefficients and standard errors for the Free Choice condition model.*

| Dimension        MP                        β (SE) b (SE)                    P Value (β /b) |
| --- |
| Dimension 1                           Path Length          0.213 (0.078) 0.213(0.075)              0.006/0.004                           Normalized Path Length 0.069 (0.067)                 0.004 (0.004) 0.303/0.305                           Elliptical Area          0.827 (0.051) 0.002 (0.000)              0.000/0.000                           Error 1 Variance       0.167 (0.047) 0.001 (0.000)              0.000/0.000  Dimension 2                           Path Length          0.353 (0.159) -0.012 (0.006)            0.026/0.033                           Normalized Path Length -0.133 (0.141)                 -0.002 (0.001) 0.346/0.393                            Error 2 Variance      0.844 (0.122) 52.447 (21.645)         0.000/0.000 |

Note: MP = Movement Parameter, b = Unstandardized Estimate, β = Standardized Estimate and SE = Standard Error

Table S4. *Covariance matrix for the variables included in the Forced Scale condition model.*

| DIM1 DIM2 PATHL ELIP PATHN |
| --- |
| DIM1 1.597  DIM2 -0.017 0.403  PATHL -26.477 11.602 10361.654  ELIP -1.735 2.067 1002.730 120.490  PATHN -2.143 -5.305 814.388 -0.098 1.00 |

Note: DIM1 = Dimension 1, DIM2 = Dimension 2, ELIP = Elliptical Area, PATHL = Path Length and, PATHN = Normalized Path Length

*Table S5.* The values of the fit indices for the Forced Scale condition model.

| Fit Index                                     Fit Index Value                  Fit Indicated |
| --- |
| RMSEA                                            0.000                  Excellent Fit  CFI                                                   1.000              Excellent Fit  TLI                                                   1.000              Excellent Fit  SRMR                                              0.008                   Close Fit |

Table S6*.* *Standardized and unstandardized path coefficients and standard errors for the Forced Scale condition model.*

| Dimension        MP                        β (SE) b (SE)                        P Value (β /b) |
| --- |
| Dimension 1                           Path Length          -0.552 (0.443) -0.011(0.005)             0.046/0.045                           Normalized Path Length 0.234 (0.179)                 0.009 (0.007) 0.191/0.189                           Elliptical Area          0.692 (0.460) 0.080 (0.053)              0.132/0.132                           Error 1 Variance       0.917 (0.067) 1.464 (0.192)             0.000/0.000  Dimension 2                           Path Length          0.353 (0.159) -0.012 (0.006)            0.026/0.033                           Normalized Path Length -0.133 (0.141)                 -0.002 (0.001) 0.346/0.393                            Error 2 Variance      0.870 (0.068) 0.350 (0.038)             0.000/0.000 |

Note: MP = Movement Parameter, b = Unstandardized Estimate, β = Standardized Estimate and SE = Standard Error

Table S7. *Covariance matrix for the variables included in the Binary Choice condition model.*

| DIM1 DIM2    PATHL ELIP          PATHN |
| --- |
| DIM1            1.942  DIM2           0.001 0.058  PATHL        -26.207 0.889       10361.654  ELIP             -1.905 0.395       1002.730 120.490  PATHN        0.480 -2.409      814.388 -34.600     1041.223 |

Note: DIM1 = Dimension 1, DIM2 = Dimension 2, ELIP = Elliptical Area, PATHL = Path Length and, PATHN = Normalized Path Length

Table S8. *The values of the fit indices for the Binary Choice condition model.*

| Fit Index Fit Index Value Fit Indicated |
| --- |
| RMSEA 0.000 Excellent Fit  CFI 1.000 Excellent Fit  TLI 1.000 Excellent Fit  SRMR 0.037 Close Fit |

Note: CFI = Comparative Fit Index, RMSEA = Root Mean Square Error of Approximation, SRMR = Standardized Root Mean Square Residual, TLI = Tucker Lewis Index.

Table S9. *Standardized and unstandardized path coefficients and standard errors for the Binary Choice condition model.*

| Dimension MP β (SE) b (SE) P Value (β /b) |
| --- |
| Dimension 1  Path Length -0.896 (0.426) -0.012 (0.006) 0.035/0.034  Normalized Path Length 0.302 (0.170) 0.013 (0.007) 0.075/0.071  Elliptical Area 0.709 (0.440) 0.090 (0.056) 0.108/0.106  Error 1 Variance 0.920 (0.065) 1.786 (0.199) 0.000/0.000  Dimension 2  Path Length 0.121 (0.080) 0.000 (0.000) 0.133/0.139  Normalized Path Length -0.340 (0.094) -0.003 (0.001) 0.000/0.000  Error 2 Variance 0.890 (0.058) 0.051 (0.007) 0.000/0.000 |

Note: MP = Movement Parameter, b = Unstandardized Estimate, β = Standardized Estimate and SE = Standard Error
